# All‐Inside Anterior Cruciate Ligament Reconstruction Using Semitendinosus Autograft Augmented With Biocomposite Scaffold: A Technical Note

**DOI:** 10.1002/atn2.70292

**Published:** 2026-10-09

**Authors:** Abdullah Alanazi, Hammad Alomaim, Raghad Alaujan, Firas Alsebayel, Wazzan Aljuhani

**Affiliations:** ^1^ Department of Orthopaedic Surgery Ministry of National Guard Health Affairs Riyadh Saudi Arabia; ^2^ King Abdullah International Medical Research Center Riyadh Saudi Arabia; ^3^ King Saud Bin Abdulaziz University for Health Sciences Riyadh Saudi Arabia

## Abstract

Anterior cruciate ligament reconstruction using the all‐inside technique offers bone preservation and minimal invasiveness. However, single hamstring tendon autografts with a diameter below 8 mm carry increased failure risk. BioBrace (ConMed), a biocomposite scaffold of bioresorbable poly(L‐lactide) microfilaments and type I collagen, provides mechanical reinforcement and biological support for graft healing. The purpose of writing this article is to describe an all‐inside anterior cruciate ligament reconstruction using quadrupled semitendinosus autograft augmented with BioBrace biocomposite scaffold.

## Graphical abstract

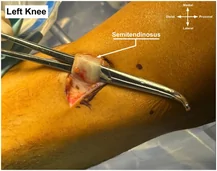

**VIDEO 1:** This video shows surgical steps in anterior cruciate ligament reconstruction using an all‐inside hamstring tendon autograft augmented with BioBrace. The graft and reconstruction follow standard procedure with great attention to BioBrace graft augmentation. Video content can be viewed at https://doi.org/10.1002/atn2.70292.

Anterior cruciate ligament (ACL) injury incidence remains high. Several techniques have been developed to improve the outcomes of anterior cruciate ligament reconstruction (ACLR). The use of all‐inside technique has gained popularity in the recent years because of its unique features in terms of bone preservation and better graft tension control, and it decreased invasiveness.^1^, ^2^ Several autograft options have been proposed for ACLR. Each graft option has its own pros and cons. The use of hamstring tendon autograft is an attractive choice owing to its low donor site morbidity and excellent patient outcomes.^3^, ^4^ With the use of all‐inside technique, a single hamstring tendon, specifically the semitendinosus, can be sufficient, because the decreased socket length preserves the gracilis tendon.^2^ This not only ensures that the main tendon remains intact but also minimizes harvest site morbidity.^5^ Nevertheless, in certain occasions, the use of 1 hamstring tendon may lead to decreased graft diameter below 8 mm which, in turn, increases the risk of subsequent graft failure.^6^ Many surgeons use internal brace to augment the graft. This may provide mechanical support to the graft but may not help in the acceleration of the healing process of the graft.^7^ The BioBrace (ConMed) is a biocomposite scaffold which measures 5 mm in thickness composed of bioresorbable poly(L‐lactide) microfilaments and highly porous type I collagen. Its biomechanical properties enable it to mechanically reinforce the graft, increasing the graft diameter and providing a biological environment to enhance healing. In this technical note, we describe the use of BioBrace to augment a semitendinosus autograft in all‐inside ACLR.

## SURGICAL TECHNIQUE

The Video 1 shows surgical steps in ACLR using an all‐inside hamstring tendon autograft augmented with BioBrace. The graft and reconstruction follow standard procedure with great attention to BioBrace graft augmentation.

### Graft Harvesting

The described operative technique is done using hamstring autograft (quadruple semitendinosus), which is harvested by the following technique: the knee is flexed at 90°, and the hamstring tendons are palpated at their attachment site on the pes anserinus 3 cm medial and 3 cm distal to the tibial tubercle. An oblique incision is around 2 to 3 cm medial to the tibial tubercle. Dissection is done carefully to protect the superficial layer of the medial collateral ligament, infrapatellar, and sartorial branches of the saphenous nerve. After incising sartorius facia, tendinous attachments of sartorius, gracilis, and semitendinosus are identified, released in the deep plane of sartorius fascia, and pulled medially using right angle forceps, as shown in Figure 1. An open loop tendon stripper (Smith & Nephew) is carefully inserted to detach the tendon from its muscle fibers, as shown in Figure 2. The length of the harvested semitendinosus is around 26 to 28 cm, whereas the length of the quadrupled semitendinosus graft is 55 to 60 mm, as shown in Figure 3.

**FIGURE 1:**
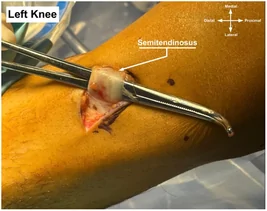
Intraoperative image of the left knee from medial viewpoint. After careful dissection at the pes anserine, the semitendinosus tendon is identified from the tibial insertion.

**FIGURE 2:**
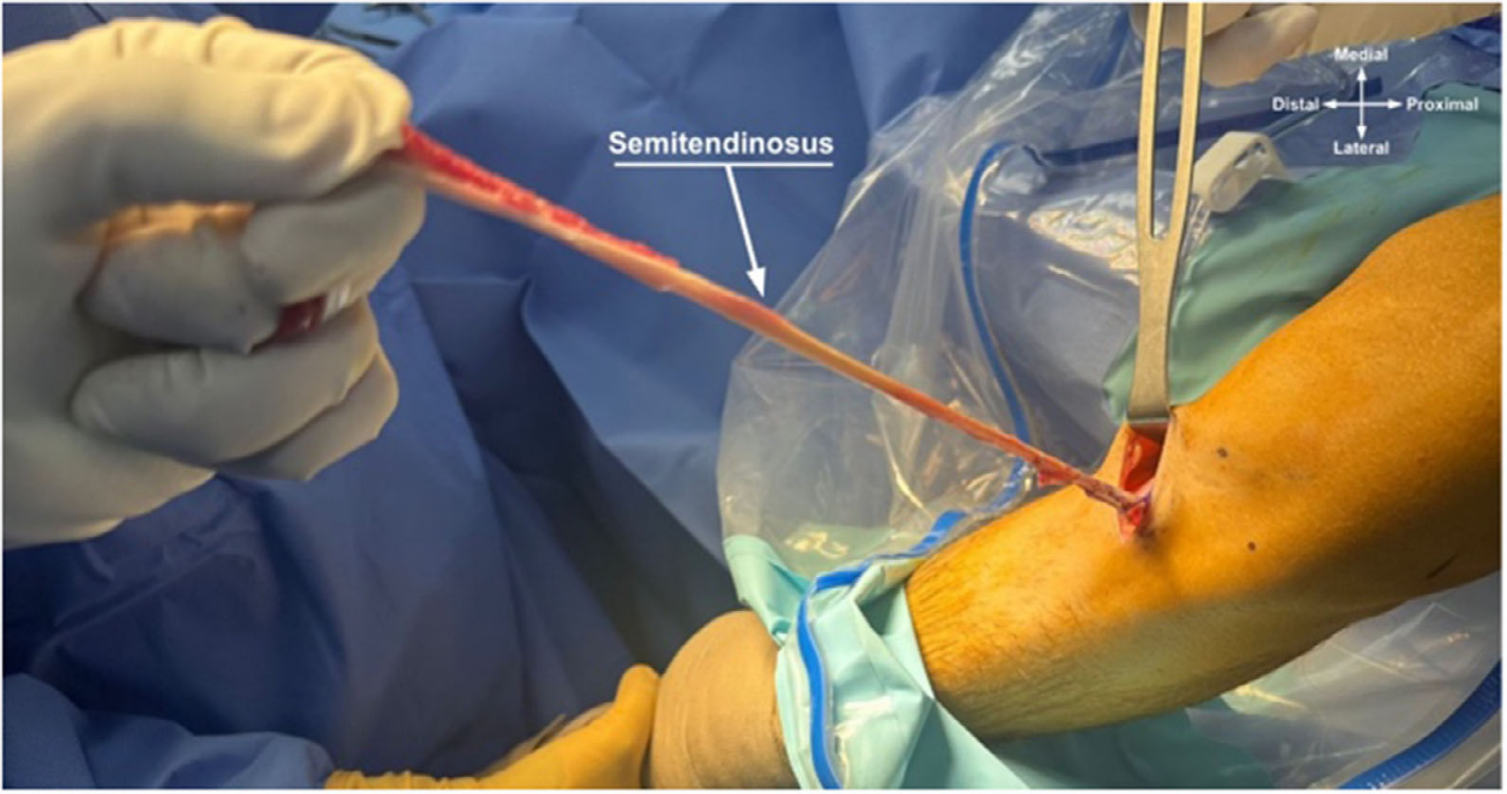
Intraoperative image of the left knee from medial viewpoint. After release from its insertion, the semitendinosus tendon is harvested with a tendon stripper.

**FIGURE 3:**
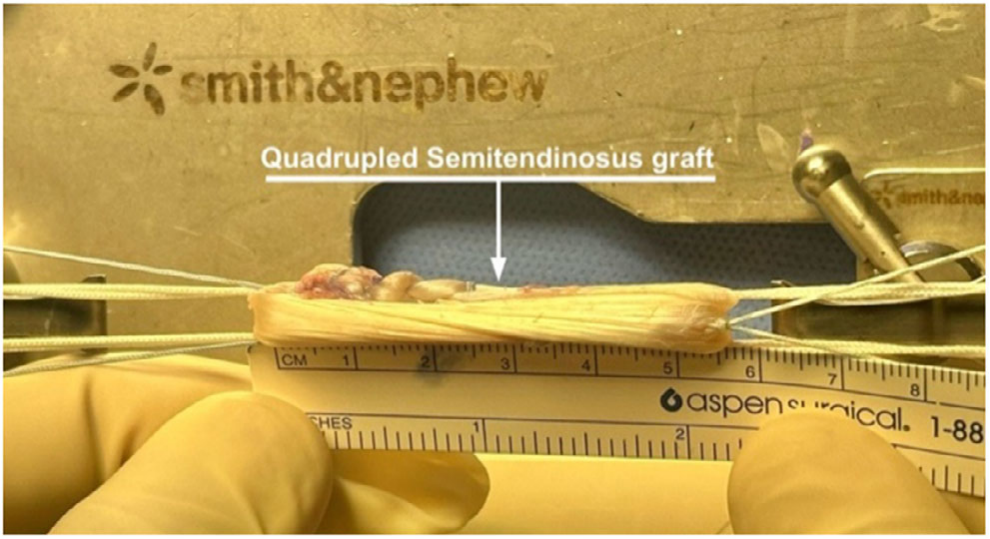
Intraoperative image of the harvested graft preparation on back table. The semitendinosus graft is quadrupled by folding twice, and the tail ends are secured with looped suture.

### Graft Preparation

The graft preparation is done on the graft preparation table (Smith & Nephew). BioBrace with a thickness of 5 mm is measured and folded to match semitendinosus autograft length. The BioBrace is hydrated for around 1 to 2 minutes with a saline solution to make it more flexible during preparation, as shown in Figure 4; then, we marked 20 mm from each end to identify the femoral and tibial socket positions. The ACL graft preparation system is used to prepare the graft. The BioBrace is centered around the graft, and then, it is fixed at 1 end using circumferential sutures. The FiberLoop (Arthrex) stitches are passed through the graft using the recommended fashion using a Keith needle, with care taken to pierce the semitendinosus graft, as well as the BioBrace with each pass. Starting 20 mm from the end of the graft, 2 passes are made toward the end of the graft. A circumferential running suture around the graft and BioBrace is used ensuring that the BioBrace is secured to the autograft. The ends of the suture are tied, and the knot is buried within the graft. The diameter of the graft is then measured, and the graft is then placed on the graft preparation station and tensioned (Figure 5).

**FIGURE 4:**
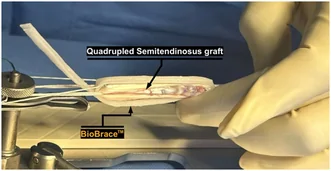
Intraoperative image of graft augmentation on back table. The BioBrace strip is wrapped on the graft under tension in preparation for graft augmentation.

**FIGURE 5:**
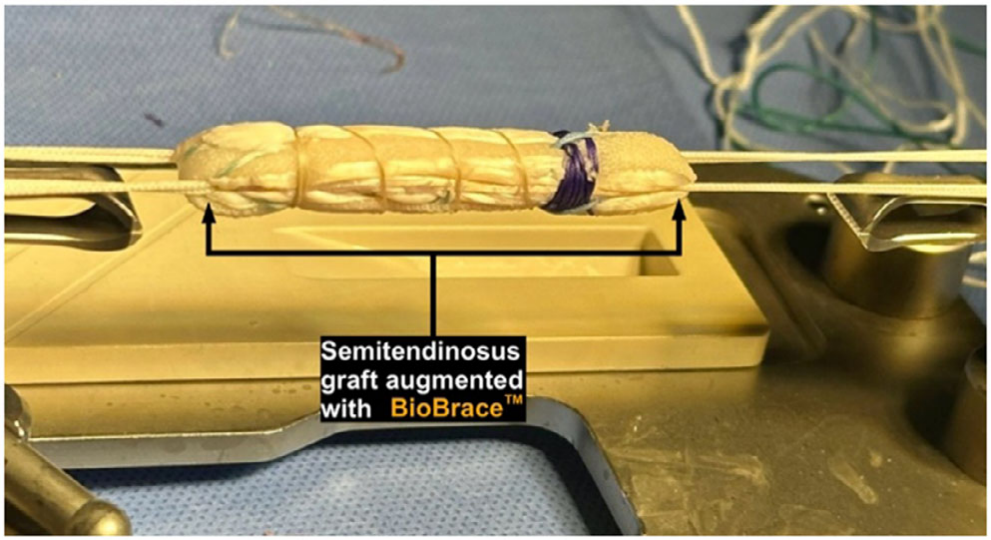
Intraoperative image of final graft augmentation on back table. The semitendinosus graft is augmented with the BioBrace under tension.

### ACL Reconstruction

Anterolateral and anteromedial portals are created, and arthroscopic examination is performed. ACL remnants from femur and tibia are preserved because it is believed that residual tissue contains mechanoreceptors which help maintain and enhance proprioception in the knee joint, as shown in Figure 6. All‐inside (ACLR, closed socket) technique is used for reconstruction. The femoral starting point is marked, and a 7‐mm transportal offset guide (Smith & Nephew) is placed at the femoral footprint. The femoral socket is created by antegrade drilling over a 2.4‐mm guide pin (Smith & Nephew) and then using a 9.5‐mm reamer (Smith & Nephew) (equal to the diameter of the graft) to create a socket over the guidewire of at least 25 mm in length, as shown in Figure 7. Then, a 4.5‐mm endobutton reamer (Smith & Nephew) is used for endobutton passage. A shuttle suture is passed through the femoral socket. The tibia guide (Smith & Nephew) is positioned 55° at the ACL footprint, as shown in Figure 8. The tibial socket is created by using a 5‐mm drill (Smith & Nephew) and then a 9.5‐mm reusable retrograde drill (SBM, Lourdes, France) with a length of 25 mm, followed by a placement of a passing suture, as shown in Figure 9.

**FIGURE 6:**
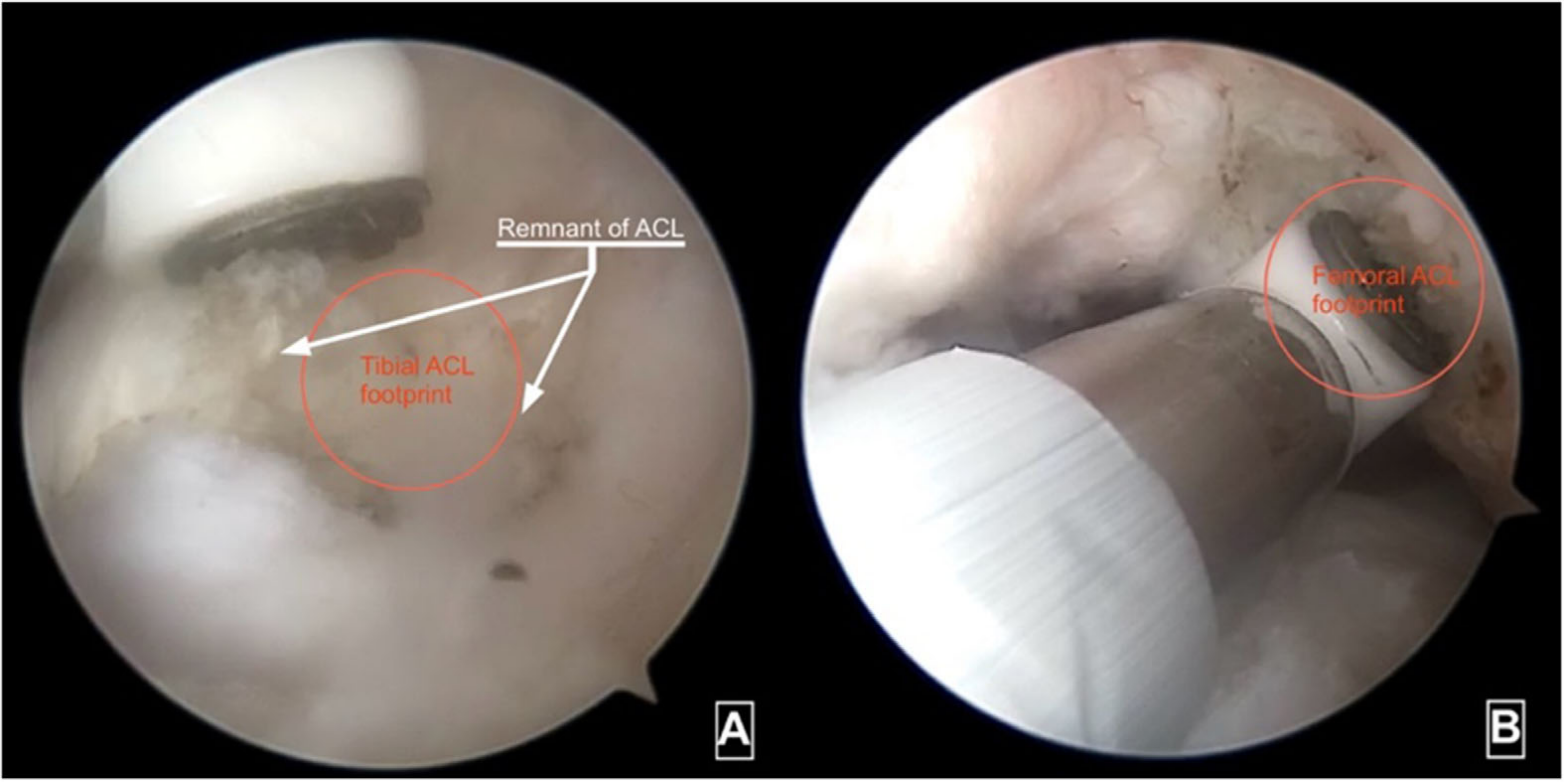
Viewing through an anterolateral portal of the left knee, ACL remnants from tibia (A) and femur (B) are preserved because it is believed that residual tissue contains mechanoreceptors which help maintain and enhance proprioception in the knee joint. (ACL, anterior cruciate ligament.)

**FIGURE 7:**
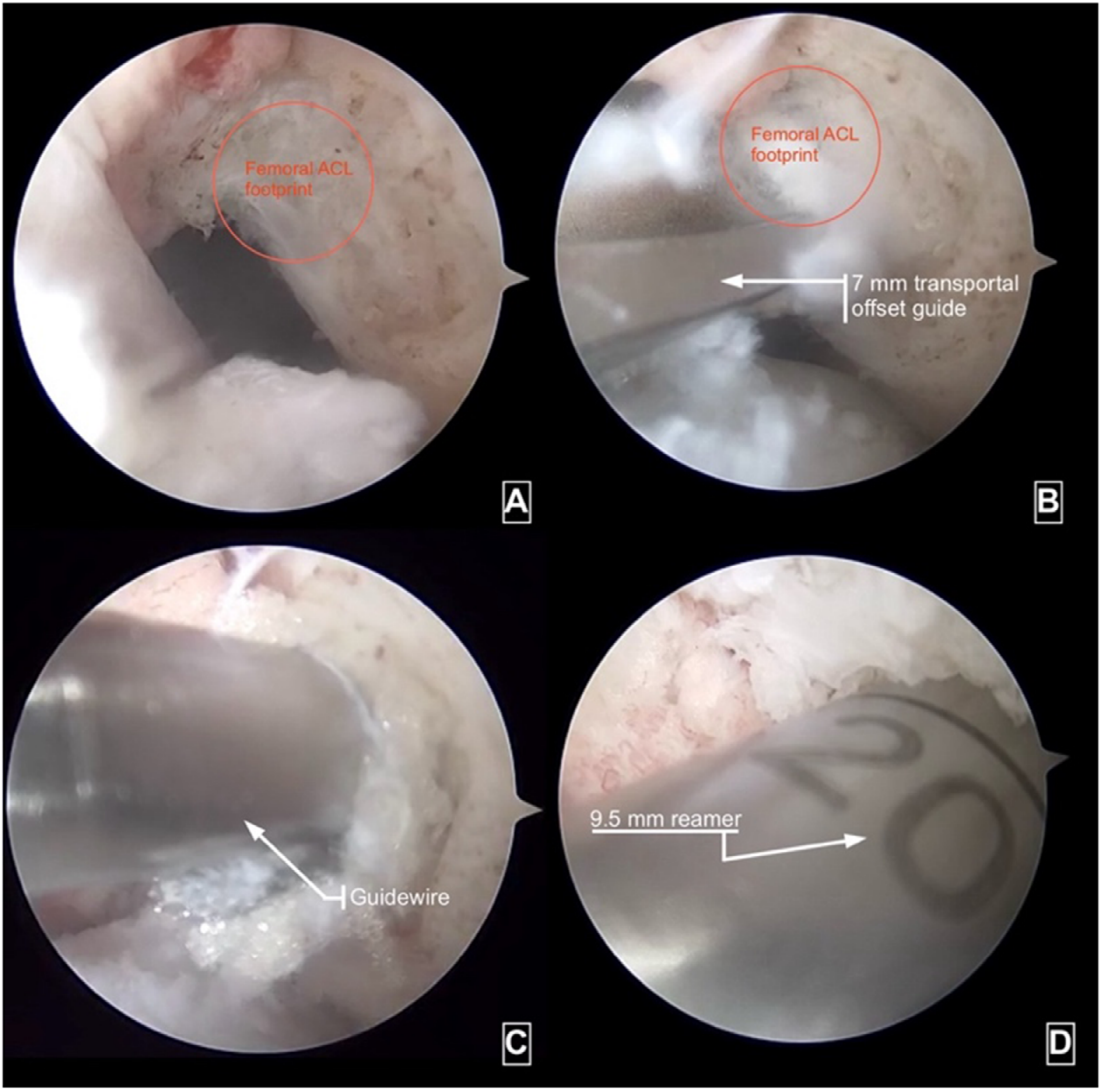
(A) Viewing through an anterolateral portal of the left knee, location of the ACL footprint. (B) The 7‐mm transportal offset guide is positioned at the anatomic location on the lateral wall of the intercondylar notch. The femoral socket is created by antegrade drilling a guide pin (C) and then using a 9.5‐mm reamer (D). (ACL, anterior cruciate ligament.)

**FIGURE 8:**
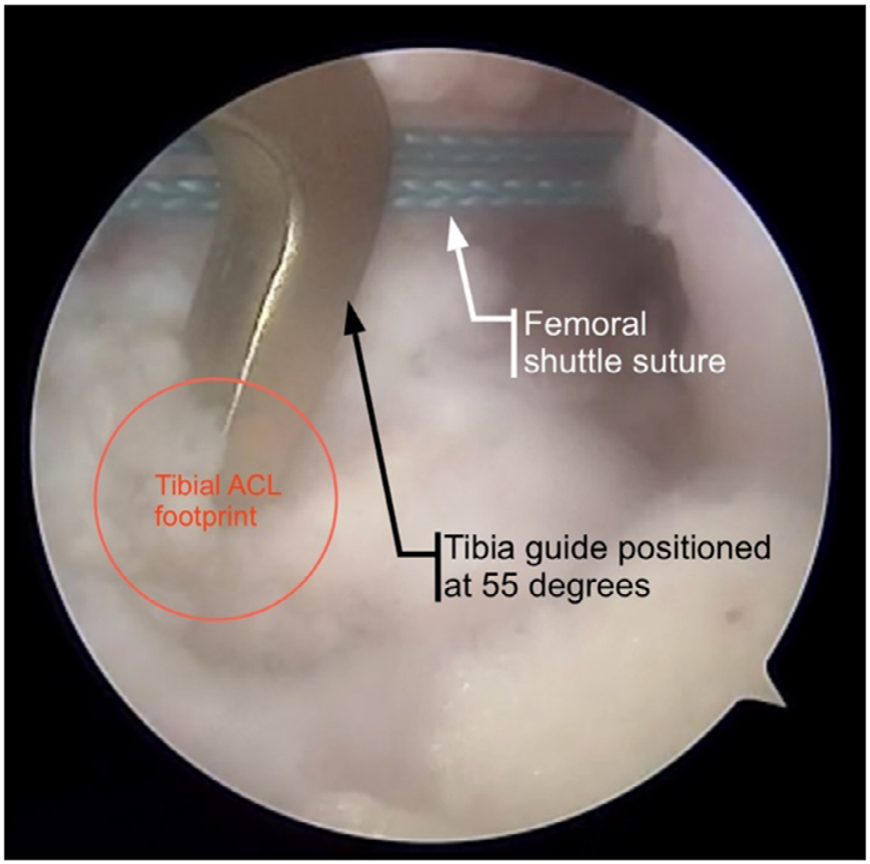
Viewing through an anterolateral portal of the left knee, showing that the tibia guide is positioned at 55° at the ACL footprint as shown. Note the femoral shuttle suture passing through the previously created femoral socket. (ACL, anterior cruciate ligament.)

**FIGURE 9:**
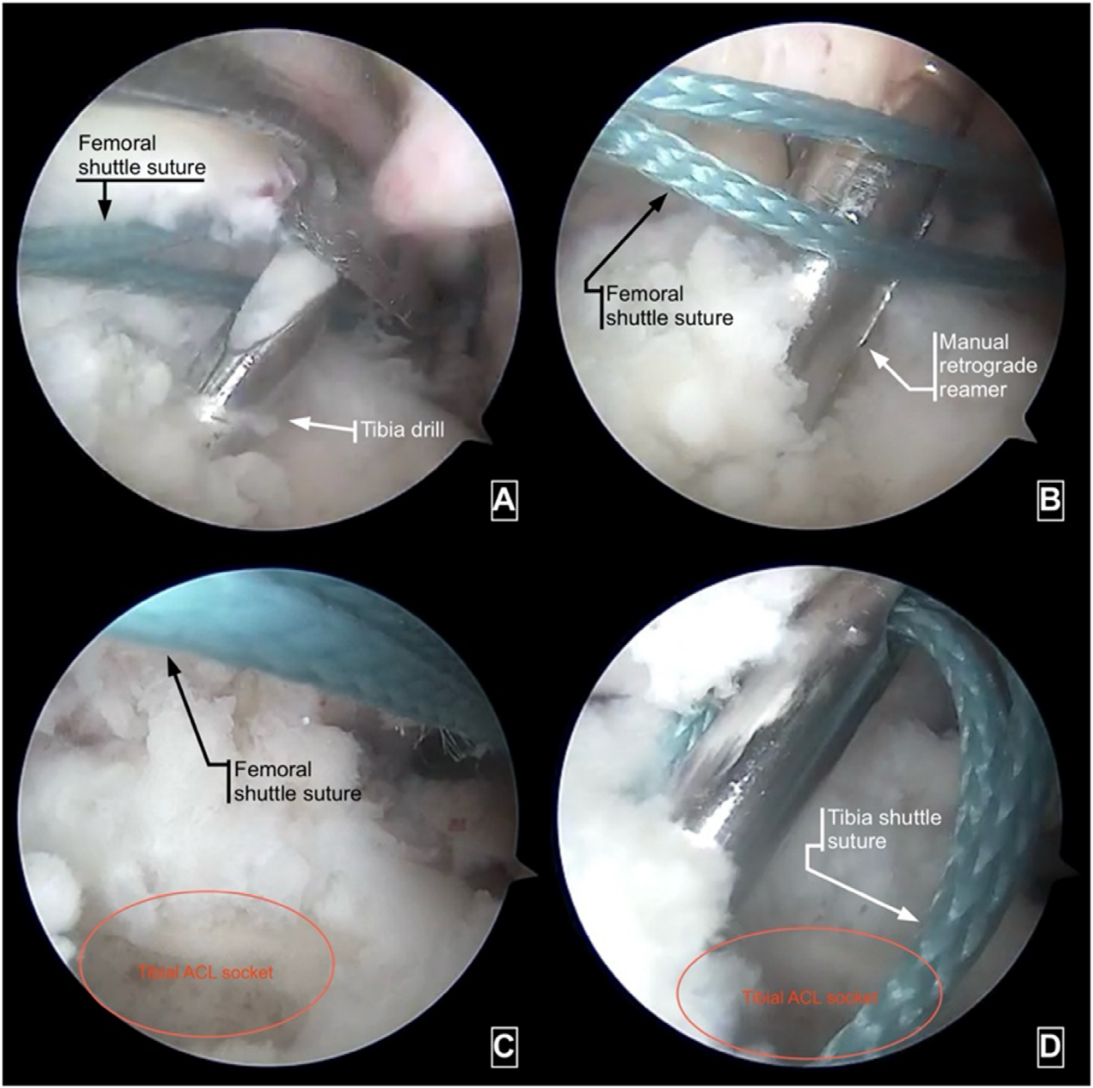
Viewing through an anterolateral portal of the left knee. (A) The tibial socket is created by using a 5‐mm drill. (B) Then, a 9.5‐mm reusable retrograde drill with a length of 25 mm is used (equal to the diameter of the graft) to create the tibial socket. (C) Tibial ACL socket after reaming shown. (D) Tibial shuttle suture is passed through the previously created tibial socket. (ACL, anterior cruciate ligament.)

### Graft Passage

The passing sutures of femoral and tibial are pulled out through the anteromedial portal, with care to avoid any soft‐tissue bridge, as shown in Figure 10. The femoral Pullup endobutton (SBM, Lourdes, France) is pulled up through the femoral socket and flipped to ensure that it rests flushed on the femoral cortex, as shown in Figure 11. The femoral side of the graft is pulled into the socket approximately 20 mm. The tibial‐sided sutures are pulled out of the tibial socket, and the graft is docked into the tibial socket. Then, tibial fixation is done via Pullup endobutton (SBM, Lourdes, France) on the anteromedial tibial cortex, and the tibial Pullup sutures are shortened until fully seated. After cycling the knee, final tension is applied to the graft on both ends of the femoral and tibial sides at 30° knee flexion, the sutures are tied with square knots, and the augmented graft has been securely implanted within the knee, as shown in Figures 12 and 13.

**FIGURE 10:**
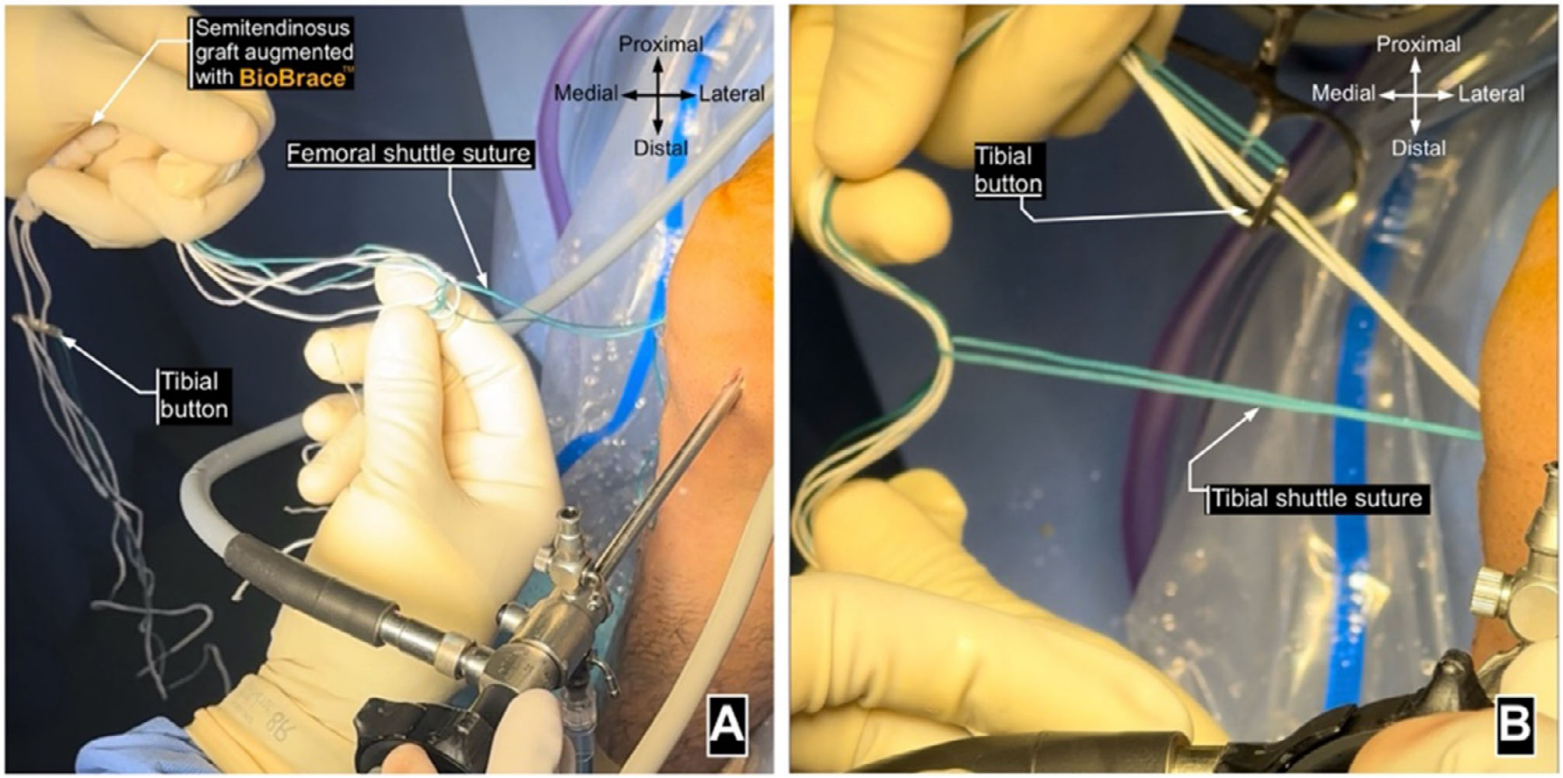
Intraoperative image of the left knee. The passing sutures of the femoral (A) and tibial (B) are pulled out through the medial portal.

**FIGURE 11:**
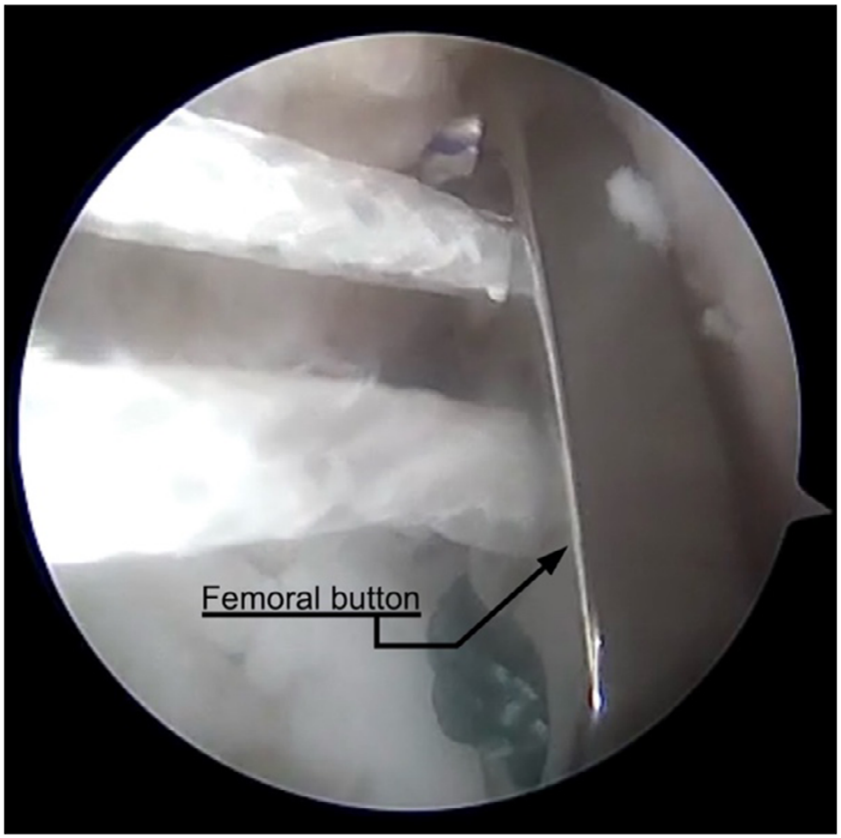
Viewing through an anterolateral portal of the left knee, showing the femoral endobutton being pulled up through the femoral socket and flipped to ensure that it rests flushed on the femoral cortex.

**FIGURE 12:**
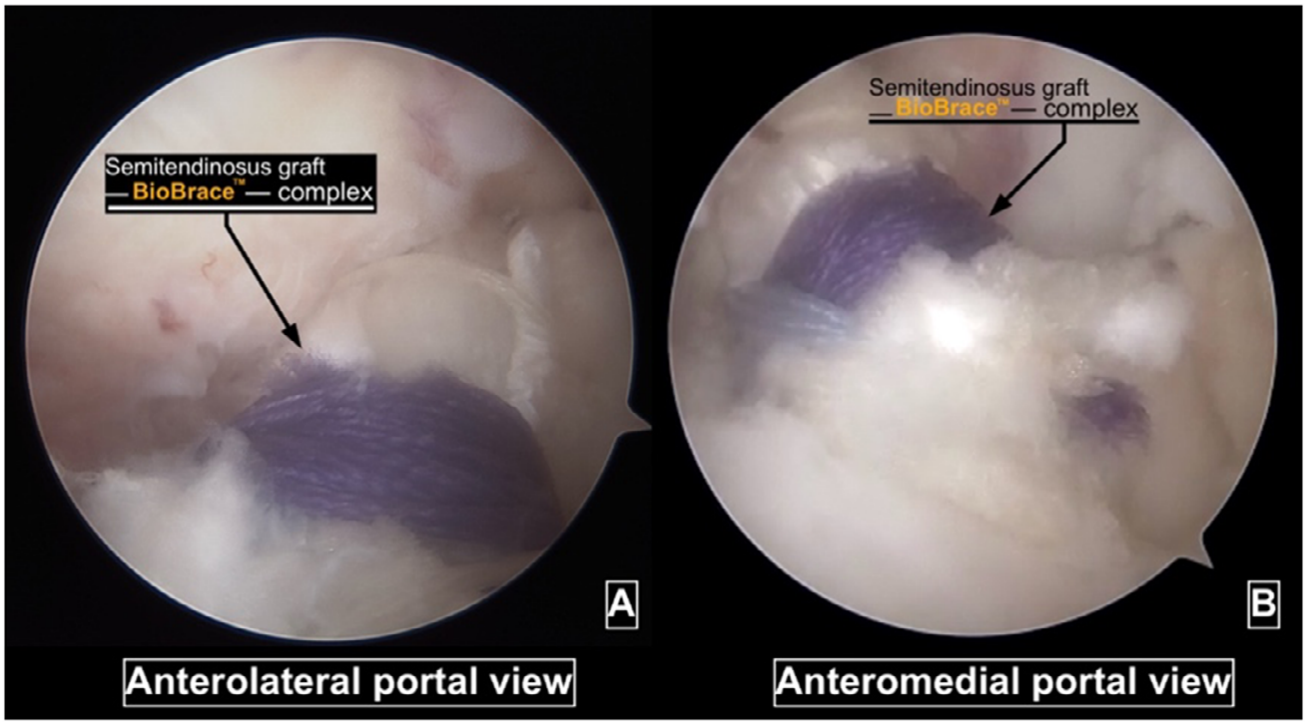
Viewing through an anterolateral (A) and anteromedial (B) portal of the left knee showing graft position after final tensioning to the graft on both ends of the femoral and tibial sides.

**FIGURE 13:**
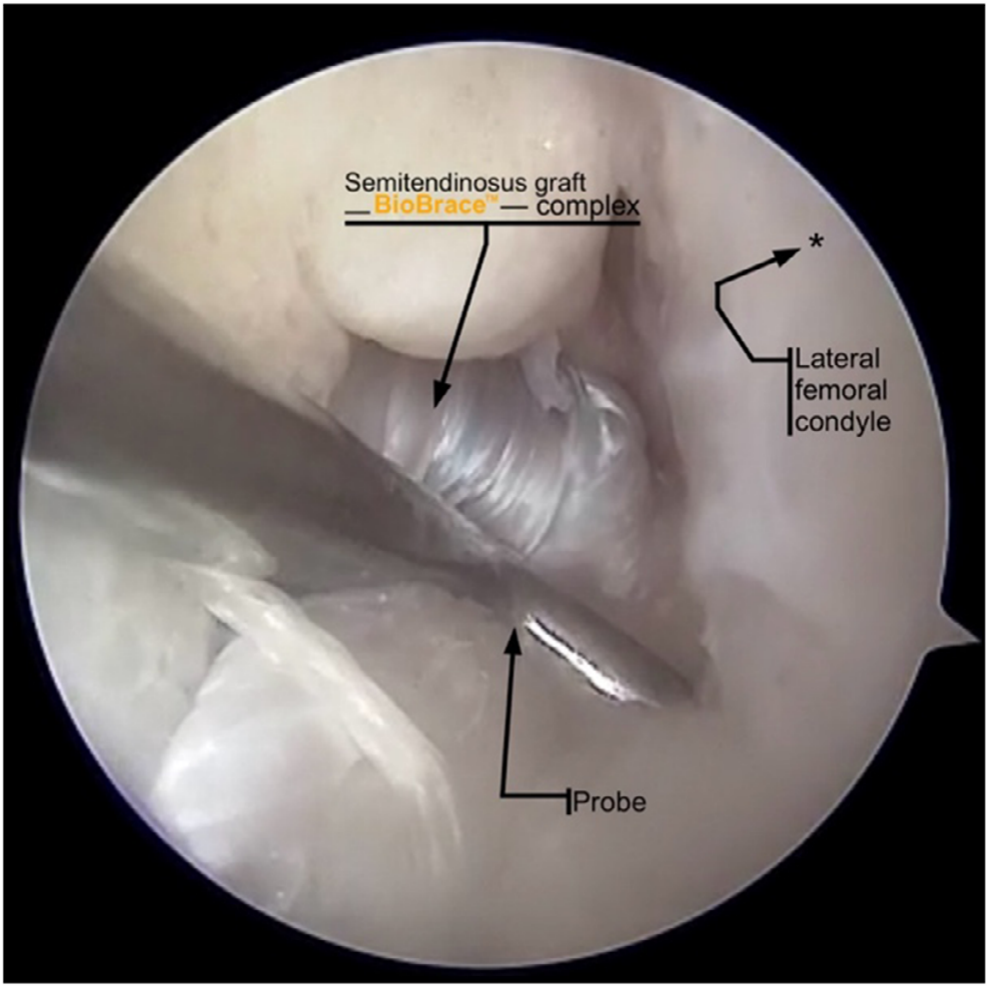
Arthroscopic image of the left knee through anterolateral portal. After placement and tensioning, the graft is inspected with a probe to check for impingement on the lateral femoral wall or posterior cruciate ligament.

### Rehabilitation Protocol

Postoperative rehabilitation is implemented as the typical ACLR protocol without any modifications because of the use of BioBrace. Patients are allowed early weight‐bearing as tolerated if ACL is reconstructed without meniscus repair or cartilage procedure. Progressive range of motion exercises begins early with focus on restoring full extension and enhancing quadriceps recovery. Return to sport is not earlier than 9 months after clearance from orthopaedic surgeon and physiotherapist.

## DISCUSSION

ACL injury incidence remains high. Several techniques have been developed to improve the outcomes of ACLR. The all‐inside technique, which gained popularity in recent years, offers unique features such as bone preservation, better graft tension control, and reduced invasiveness. In this surgical technique paper, we described the all‐inside technique using semitendinosus autograft augmented with BioBrace biocomposite scaffold. The motivation behind augmentation is the belief that it can enhance the remodeling and biological integration of the graft, as well as provide some early mechanical strength to the graft. The BioBrace biocomposite scaffold provides immediate mechanical reinforcement by load‐sharing approximately 140 N at implantation, which can compensate for the early weakness of autografts or undersized allografts and reduce elongation during the “ligamentization” phase.^8^ By suturing the porous type I collagen‐PLL microfilament strip to the graft, the construct gains 1 to 2 mm in diameter, addressing the well‐documented risk of failure in hamstring tendons <8 mm and improving postoperative strength and stability. Moreover, the highly porous architecture supports fibroblast infiltration and collagen maturation, accelerating graft‐bone integration as evidenced by magnetic resonance imaging‐graded grade I healing at 18 months in the hamstring‐BioBrace case.^9^ Despite these benefits, several limitations warrant consideration. The implant adds around 0 to 15 minutes to graft preparation and requires meticulous handling to avoid scaffold cutout, thereby increasing operative time and technical complexity.^10^ The off‐the‐shelf device carries a higher material cost, which may affect health economic decisions, especially given the current lack of long‐term comparative data on retear rates and functional outcomes.^10^, ^11^, ^12^, ^13^ Additionally, although the scaffold resorbs biologically, the timing of degradation must match tissue remodeling to prevent premature loss of support or stress shielding, a balance not yet fully validated in large‐scale cohorts.^14^ In summary, BioBrace augmentation offers a compelling combination of early mechanical support and biologic facilitation of graft incorporation, but its increased cost, operative demands, and limited long‐term evidence should be weighed against patient‐specific factors such as graft size, activity level, and risk profile. Further randomized trials are needed to define its optimal indications and confirm durability. The advantages and disadvantages are listed in Table 1, whereas the pearls and pitfalls are listed in Table 2.

**TABLE 1:** BioBrace (Biocomposite Collagen‐Poly‐L‐Lactide Scaffold): Advantages Versus Disadvantages

Advantages	Disadvantages
Early load‐sharing strength (140 N tensile load at implantation)	Higher implant cost
Improves graft‐bone integration and biologic healing (collagen matrix and resorbable PLLA)	Slightly longer graft preparation time around 2‐3 min
Increases graft diameter by 1‐2 mm, which is useful for small‐diameter hamstring or quadriceps grafts	Limited long‐term clinical outcome data because there are no studies with clinical outcome duration more than 2 yr
Compatible with autograft, allograft, or soft‐tissue grafts (BPTB, hamstring, quadriceps)	Potential for increased infection risk
Poly‐L‐lactide microfilaments gradually resorb as native tissue remodels	Possible inflammatory response to PLLA microfilaments
Low donor‐site morbidity (no extra harvest needed)	Risk of overtightening or oversizing leading to notch impingement or cyclops lesion
Simple “sandwich” suturing with FiberTag/TightRope system; reproducible technique	Requires additional assistant or equipment and careful handling to avoid scaffold damage

BPTB, bone–patellar tendon–bone; min, minutes; PLLA, poly‐L‐lactic acid; yr, years.

**TABLE 2:** Pearls and Pitfalls of BioBrace for Graft Augmentation

Pearls	Pitfalls
Uniform “cable‐wrap” or “sandwich” placement of the scaffold	Oversizing the graft‐scaffold construct (adding >2 mm)
Match BioBrace length precisely to the graft (±2 mm) and mark 20 mm from each end	Incomplete trimming of scaffold edges result in “flared” ends
Hydrate the scaffold in saline (or PRP/BMAC) before handling	Failure to keep the scaffold uniformly wrapped
Place the BioBrace on the opposite side of the FiberTag suture tape for optimal tabularization	Using a cutting needle or too close to scaffold border when suturing
Use a small‐bore, noncutting needle (e.g., CT‐2) and 2‐0 heavy braided suture to secure the scaffold	Delayed or inadequate hydration of the BioBrace
Record intraoperative graft tension (final ≈ 20 N) before fixation	Insufficient assistant/equipment readiness at case start
Verify final graft‐scaffold diameter fits comfortably within the retrograde sockets (9‐10 mm)	Relying solely on BioBrace for mechanical support (ignoring need for proper fixation)
Maintain a clean, dry field when handling the scaffold to preserve its porous structure	Not verifying final graft‐scaffold diameter before socket preparation

BMAC, bone marrow aspirate concentrate; PRP, platelet‐rich plasma.

## DISCLOSURES

The authors (A.A., H.A., R.A., F.A., W.A.) declare that they have no known competing financial interests or personal relationships that could have appeared to influence the work reported in this article.

## References

[1] Pautasso A , Capella M , Barberis L , Drocco L . All‐inside technique in ACL reconstruction: Mid‐term clinical outcomes and comparison with AM technique (hamstrings and BpTB grafts). Eur J Orthop Surg Terminol. 2021;31:1025‐1032. 10.1007/s00590-020-02798-w PMC7981303 32936315

[2] Connaughton AJ , Geeslin AG , Uggen CW . All‐inside ACL reconstruction: How does it compare to standard ACL reconstruction techniques? J Orthop. 2017;14:501‐505. 10.1016/j.jor.2017.03.002 PMC5360217 28360487

[3] Kay J , Liotta ES , Sugimoto D . Assessment of return to sport after ACL reconstruction with soft tissue autograft in adolescent athletes: Quadriceps versus hamstring tendon. Orthop J Sports Med. 2023;11:23259671231207113. 10.1177/23259671231207113 PMC10676070 38021299

[4] Hardy A , Casabianca L , Andrieu K , Baverel L . Complications following harvesting of patellar tendon or hamstring tendon grafts for anterior cruciate ligament reconstruction: Systematic review of literature. Orthop Traumatol Surg Res. 2017;103:S245‐S248. 10.1016/j.otsr.2017.09.002 28888527

[5] Tanpowpong T , Kuptniratsaikul S . Maximizing graft diameter: Unleashing the power of triple‐fold hamstring autografts for anterior cruciate ligament reconstruction. Arthrosc Tech. 2024;13:e1001‐e1005. 10.1016/j.eats.2024.103131 PMC11704893 39780883

[6] Conte EJ , Hyatt AE , Gatt Jr CJ , Dhawan A . Hamstring autograft size can be predicted and is a potential risk factor for anterior cruciate ligament reconstruction failure. Arthroscopy. 2014;30:882‐890. 10.1016/j.arthro.2014.03.028 24951356

[7] Costa GG , Perelli S , Grassi A , Russo A . Minimizing the risk of graft failure after anterior cruciate ligament reconstruction in athletes. A narrative review of the current evidence. J Exp Orthop. 2022;9:62. 10.1186/s40634-022-00461-3 PMC8924335 35292870

[8] Ozonoff A , Viswanathan G . ACL reconstruction augmented with a novel reinforced bioinductive implant: A case report with 18‐month follow‐up. J Orthop Exp Innov. 2025;6:1‐5.

[9] Mariscalco MW , Flanigan DC , Mitchell J . The influence of hamstring autograft size on patient‐reported outcomes and risk of revision after anterior cruciate ligament reconstruction: A multicenter orthopaedic outcomes network (MOON) cohort study. Arthroscopy. 2013;29:1948‐1953. 10.1016/j.arthro.2013.08.025 PMC3844091 24140144

[10] Pyrz K , Wood A , Campbell C , Brabston E , Evely T . Anterior cruciate ligament reconstruction with quadriceps tendon autograft: Surgical technique using augmentation with a biocomposite scaffold. Arthrosc Tech. 2023;12:e1101‐e1107. 10.1016/j.eats.2023.08.003 PMC10773234 38196875

[11] Marks Benson E , Pyrz K , Wood A . Anterior cruciate ligament reconstruction using bone‐tendon‐bone allograft: Surgical technique using augmentation with bio‐composite scaffold. Arthrosc Tech. 2024;13:e245‐e250. 10.1016/j.eats.2023.11.005 PMC10995643 38584643

[12] Serour P , Oladeji LO , Nuelle CW , DeFroda SF . Preparation of bone patellar tendon bone allograft with biocomposite scaffold augmentation. Arthrosc Tech. 2024;13:e1007‐e1012. 10.1016/j.eats.2024.103120 PMC11662886 39711895

[13] Bi AS , Hughes AJ , Savage‐Elliott I . Anterior cruciate ligament allograft reconstruction augmented with a reinforced, bioinductive collagen scaffold in the setting of multiligamentous knee injury. Arthrosc Tech. 2024;13:e201‐e206. 10.1016/j.eats.2023.09.034 PMC10907961 38435259

[14] Gustin MJ , Sing DC , Abdul‐Rassoul H . Return to sport after ACL reconstruction with a BTB versus hamstring tendon autograft: A systematic review and meta‐analysis. Orthop J Sports Med. 2020;8:2325967120964919. 10.1177/2325967120964919 PMC7745570 33403206

